# Preoperative activity, postoperative flexion contractures, and degree of medial cartilage damage affects achievement of high physical activity after open wedge high tibial osteotomy

**DOI:** 10.1002/jeo2.70372

**Published:** 2025-07-24

**Authors:** Fumiyoshi Kawashima, Jun Oike, Kazuyuki Segami, Koji Kanzaki

**Affiliations:** ^1^ Department of Orthopaedic Surgery Showa University Fujigaoka Hospital Yokohama Japan

**Keywords:** correction angle, high tibial osteotomy, Knee Injury and Osteoarthritis Outcome Score, preoperative medial cartilage damage, sports activities

## Abstract

**Purpose:**

To investigate factors that affect return to physical activities after open wedge high tibial osteotomy (OWHTO), and to determine whether an optimal correction angle exists for return to physical activities.

**Methods:**

Patients with medial osteoarthritis of the knee who underwent OWHTO at our institution were evaluated. Radiographic evaluations were performed using bilateral weight bearing long leg radiographs. The clinical evaluation consisted of the Tegner activity scale, the Knee Injury and Osteoarthritis Outcome Score (KOOS) Sports/Rec subscore, and the presence of residual flexion contracture of 10° or more in unstable hinge fractures. In addition, the degree of cartilage damage in the medial compartment was evaluated via arthroscopic surgical findings according to the ICRS classification.

**Results:**

Sixty patients (70 knees; 25 males and 45 females; mean age, 55.2 [32–75] years; mean follow‐up, 8.5 [3.8–12.4] years) were included in the study. The preoperative alignment defined by mean WBLR (%) was significantly higher in the Return to High Physical Activity Group (H Group: 31.0 ± 18.6) than the Return to Low Physical Activity Group (L Group: 15.9 ± 13.9). Logistic analysis showed that low preoperative WBLR and the absence of severe cartilage damage to the medial compartment, postoperative flexion contracture, and unstable hinge fracture were factors affecting return to sport. In addition, the cutoff values using the Youden Index based on ROC analysis were preoperative Tegner activity score of 4.0 and preoperative KOOS (Sports/Rec) of 35.0.

**Conclusion:**

Preoperative KOOS (Sports/Rec) was a useful index for predicting postoperative return to high physical activities. Depending on the degree of arthroscopic cartilage damage, the under‐collection of cases with severe cartilage damage should be avoided.

**Level of Evidence:**

Level IV, retrospective case series.

**Abbreviations**
ESSKA: European Society for Sports Traumatology
EZR: Easy R software
ICRS: International Cartilage Repair Society
KJLO: knee joint line orientation
KL: Kellgren–Lawrence
KOOS: Knee Injury and Osteoarthritis Outcome Score
MFC: medial femoral condyle
mLDFA: mechanical lateral distal femoral angle
MMPRT: medial meniscus posterior root tear
mMPTA: mechanical medial proximal tibial angle
MTP: medial tibial plateau
OWHTO: open wedge high tibial osteotomy
ROC: receiver‐operating characteristic
SONK: spontaneous osteonecrosis of the knee
WBLR: weight‐bearing line ratio

## INTRODUCTION

Previous reports have described satisfactory outcomes for open wedge high tibial osteotomy (OWHTO) as a treatment for highly active young patients with medial compartment osteoarthritis (OA) [1, 5, 9, 10].

Recent studies on return to physical activities after HTO have shown that early weight bearing is possible due to strong and stable fixation internal fixation implants and surgical techniques, with good return rates of 88.6% [18], 82% [9] and 89.6% [14]. However, the criteria used to define the level of exercise necessary for returning to physical activity vary widely in the literature. Although the latest consensus report from the European Society for Sports Traumatology (ESSKA) states that most patients return to a higher level as compared to their preoperative period, patients are less likely to achieve an RTS at the level they enjoyed prior to the onset of symptoms [26]. Current reports on factors that affect return to physical activities include preoperative activity [9, 12, 13], patient motivation and expectations [2, 32], body mass index (BMI) and age [11], correction angle [25], preoperative OA and degree of varus, postoperative rate of bone union [40], timing of weight bearing [30, 33], and postoperative alignment and joint line obliquity [24]. In terms of the appropriate postoperative alignment and articular slope for returning to high physical activities, neither excessive valgus [25, 38] nor insufficient correction [19, 20] is considered favourable. The purpose of this study was to hypothesise and examine whether the presence of unstable hinge fractures, severe cartilage damage of the medial component, among other indicators of preoperative activity are poor prognostic factors for returning to high physical activities.

## MATERIALS AND METHODS

### Patient selection and exclusion criteria

This retrospective observational case series was approved by this study was approved by the Ethics Committee of Showa University Fujigaoka Hospital (approval number: 2024‐248‐A). Those who underwent OWHTO for medial knee osteoarthritis at our department between January 2012 and December 2019 were evaluated in this study. A total of 142 knees underwent initial OWHTO. Our indications for OWHTO were (1) symptomatic isolated medial OA (Kellgren–Lawrence [KL] classification system; [15] KL Grade ≤ Ⅲ) or mild spontaneous osteonecrosis of the knee (SONK) (Koshino classification system; [16] Stage ≤ Ⅱ) with varus malalignment of 2°–10°, (2) range of motion of 120° or more of flexion and < 20° of extension loss, and (3) absence of advanced patellofemoral OA. Patients with postoperative infection, cruciate ligament injury, bilateral OWHTO (*n* = 23), history of previous knee surgery, SONK (Koshino classification system; Stage Ⅲ or higher), and follow‐up period of less than 1 year were excluded. After exclusions, 60 knees of 60 patients were included. The sample size of this study was not designed in advance; therefore, a post hoc analysis was performed. The significance level was set to 5%, and the standard deviation value of all cases obtained within the study was used to calculate the pooled standard deviation. Easy R (EZR; Jichi Medical University) was used for software analysis. The post hoc power of each study was as follows: age, 3%; correction angle, 92%; preoperative WBLR, 84%; preoperative mechanical medial proximal tibial angle (mMPTA), 27%; preoperative mechanical lateral distal femoral angle (mLDFA), 43%; preoperative knee joint line orientation (KJLO), 31%; postoperative WBLR, 33%; postoperative mMPTA, 28%; postoperative KJLO, 35%; preoperative Knee Injury and Osteoarthritis Outcome Score (KOOS), 100%; preoperative Tegner activity score, 100%; flexion contractures (+.−) 100%, unstable hinge fractures (+.−) 24%; severe cartilage damage (+.−) 100%; postoperative OA progression in the PF joint, 4%; postoperative KOOS, 100%; postoperative Tegner activity score, 100%.

**Figure 1:**
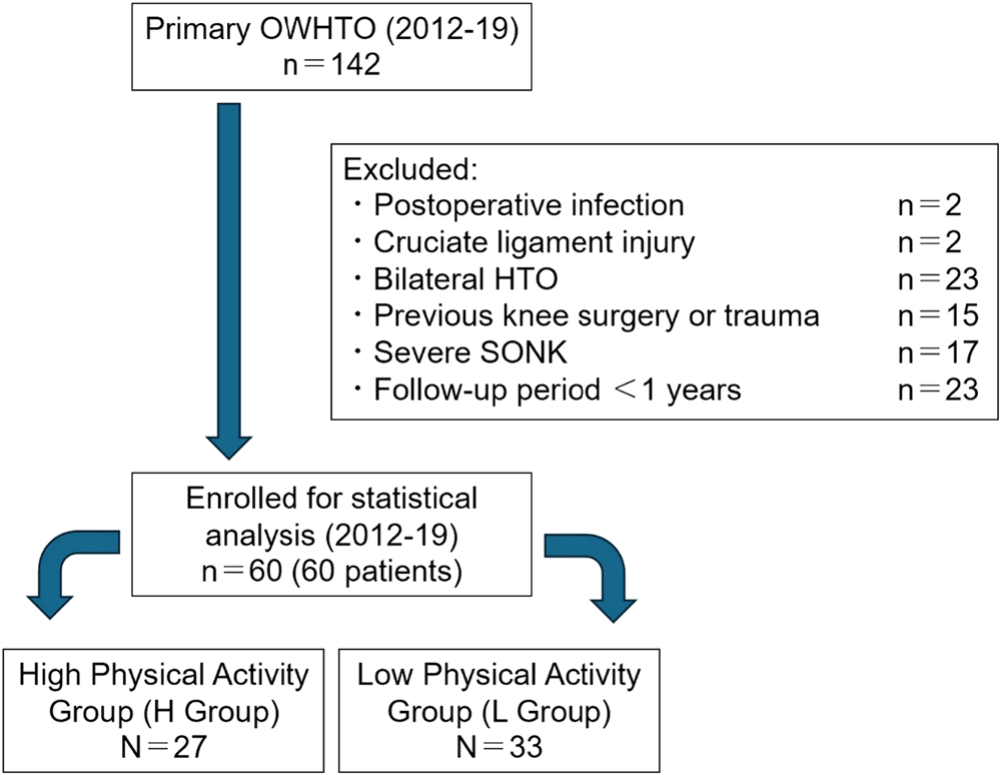
Flow chart of patient selection. HTO, high tibial osteotomy; OWHTO, open wedge high tibial osteotomy; SONK, spontaneous osteonecrosis of the knee.

The criteria for return to physical activities after surgery were based on the Tegner activity scale [36]. Patients with a Tegner scale score of 5 or higher one year after surgery were classified as the Return to High Physical Activity Group (H Group, *n* = 27: 45%), and patients with a score of 4 or lower were classified as the Return to Low Physical Activity Group (L Group, *n* = 33: 55%) (Figure 1). Previous reports on Japanese patients have set a Tegner activity score of 5 as the standard for returning to high physical activities [8, 9]. Many patients who undergo surgery are middle‐aged or elderly, and a score of 6 or above includes sports such as badminton, handball, and basketball, which are not intended as post‐operative goals for middle‐aged or elderly patients. Therefore, a score of 5 was used as the classification standard.

The degree of OA on plain radiographs was evaluated using the KL classification [15], with 14 knees classified as KL 1, 40 as KL 2, and 6 as KL 3. There were 27 patients in the High Physical Activity Group (Tegner scale score of 5 or higher one year after surgery; hereinafter referred to as H Group), and 33 patients in the Low Physical Activity Group (score of 4 or lower; hereinafter referred to as L Group) (Table 1).

**Table 1:** Demographic and clinical data of the series.

Characteristics	Total
Number of participants (*N*)	**60 knees of 60 patients**
Sex (male/female)	Male: 20; female: 40
Side (left/right)	Left: 36; right: 24
Mean age (range)	55.4 ± 9.1 (32‐75)
OA grade (KL Grade Ⅰ/Ⅱ/Ⅲ)	14/40/6 knees
Correction angle	10.0 ± 2.1
Postoperative Tegner Activity Score (5 or higher/4 or lower)	27 (5 or higher)/33 (4 or lower) knees

Abbreviations: KL, Kellgren–Lawrence; OA, osteoarthritis.

The mean age was 51.9 ± 10.7 years in the H Group and 60.0 ± 7.6 years in the L Group, and the correction angle was 8.9 ± 2.0° in the H Group and 10.9 ± 1.7° in the L Group. Both were significantly higher in the L Group (Table 2).

**Table 2:** Radiographic data.

Variables	Total
Preoperative WBLR (%)	**23.0** ± **15.72**
Postoperative WBLR (%)	58.9 ± 6.22
Preoperative mMPTA	83.8 ± 1.74
Postoperative mMPTA	91.9 ± 1.95
Preoperative mLDFA	87.7 ± 1.4
Preoperative KJLO	1.4 ± 1.7
Postoperative KJLO	1.9 ± 1.4

Abbreviations: KJLO, knee joint line orientation; mLDFA, mechanical lateral distal femoral angle; mMPTA, mechanical medial proximal tibial angle; WBLR, weight‐bearing line ratio.

### Radiographic evaluation

The lower limb alignment was evaluated in a full weight‐bearing standing position (bilateral weight‐bearing long leg radiographs), and the mMPTA: the angle between the tibial functional axis and the tibial plateau, mLDFA: the angle between the femoral functional axis and the tibial plateau [28], KJLO: the angle between the floor and the tangent of the medial/lateral tibial condyles [14] and weight‐bearing line ratio (WBLR): the distance between the medial edge of the proximal tibia and the point at which the Mikulicz line intersects the proximal tibia, divided by the width of the tibial plateau [28] were measured preoperatively and 1 year after surgery using planning software (medi CAD, Hectec, Germany). WBLR was calculated by dividing the width from the medial edge of the tibia plateau to the weight bearing axis by the width of the tibia plateau, defined as the range between 0% of the medial border and 100% of the lateral border. In addition, the presence of unstable hinge fractures of Types 1' (dash), 2, and 3 according to the Takeuchi classification [35] was measured on plain CT images taken within 1 month after surgery. The reproducibility of measurements was evaluated by two examiners, who repeated the measurements 10 times each on separate days for the pre‐measurement values of all target images. The reproducibility was evaluated using the intraclass correlation coefficient. The reliability of the values selected by the two examiners (intra‐examiner reliability) was as follows: mMPTA: 0.971; mLDFA: 0.963; KJLO: 0.985; pre‐operative WBLR: 0.917; post‐operative WBLR: 0.923. All values demonstrated a high correlation.

### Clinical evaluation

The clinical evaluation consisted of the Tegner activity scale before and one year after surgery, the Knee Injury and Osteoarthritis Outcome Score (KOOS) [31] Sports/Rec subscore, and the presence of residual flexion contracture of 10° or more one year after surgery.

### Evaluation of cartilage damage

Intraoperative arthroscopic findings included the degree of cartilage damage in the medial compartment assessed by the International Cartilage Repair Society (ICRS) classification [37] system. Grade 4 in either the medial femoral condyle or tibial femoral condyle was defined as severe cartilage damage, and Grade 3 or less was defined as non‐severe cartilage damage. The progression of cartilage damage in the PF joint was defined as progression observed in either the patella or femoral trochlea during the initial examinations and arthroscopy‐assisted implant removal. These findings were compared between the two groups.

### Surgical procedure

The cartilage and meniscus were evaluated during surgery, using an arthroscope, and if necessary, micro‐fracturing and partial meniscectomy or meniscal repair were performed on the meniscus. All patients showed micro fractures of ICRS Grade 4. Meniscus repair was performed in six patients (four underwent medial meniscus posterior root tear [MMPRT] repair using the pull‐out method), and partial meniscectomy was performed in nine patients.

A bi‐planar WHTO was performed using the method described by Staubli [34] and Nakamura [23]. The osteotomy site was filled with Osferion60 (Olympus Terumo Biomaterial, Tokyo, Japan), and internal fixation was performed using a Tris Medial HTO plate (Olympus Terumo Biomaterial, Tokyo, Japan) or TomoFix® (DePuy Synthes Inc., West Chester, Philadelphia, USA). The angle of correction was determined based on the angle and width of correction measured via mediCAD from preoperative radiographs of the entire lower limbs. The surgery and measurements were performed by two physicians, including the first author, who were orthopaedic specialists with more than 15 years of experience. Both observers had more than 10 years of experience in periarticular knee osteotomy.

### Statistical analysis

Statistical analysis was performed using SPSS statistics ver 22.0 (IBM Japan, Ltd., Tokyo, Japan). Comparisons between the two groups were performed using unpaired two‐tailed *t*‐tests for quantitative values and two‐tailed Fisher's exact test for non‐quantitative values. Analyses of factors associated with high activity return were performed using logistic regression analyses, and cutoff values for factors associated with high‐activity return were determined using Youden index methods using receiver‐operating characteristic (ROC) curves and analyses. In all cases, the significance level was set at less than 0.05.

## RESULTS

### Patient data

Of the 142 patients who underwent primary OWHTO, a total of 60 knees of 60 patients (20 males and 40 females) with a mean age of 55.4 ± 9.1(32–75) years and an average follow‐up period of 8.3 (3.8–12.4) years were included after exclusions. The degree of OA on plain radiographs was evaluated using the KL classification system (KL classification) [15], with 14 knees classified as KL 1, 40 as KL 2, and 6 as KL 3. There were 27 patients in the H Group (Tegner scale score of 5 or higher one year after surgery), and 33 patients in the L Group (a score of 4 or lower) (Table 2).

The mean age was 51.9 ± 10.7 years in the H Group and 60.0 ± 7.6 years in the L Group, and the correction angle was 8.9 ± 2.0° in the H Group and 10.9 ± 1.7° in the L Group. Both were significantly higher in the L Group (Table 1).

### Complications

Complications were observed in 5 out of 60 cases (0.08%, all in the L Group). Three cases developed infection and underwent open irrigation, debridement, and antibiotics administration; none of these cases underwent plate removal. Bone nonunion and loss of correction due to unstable hinge fractures were observed in two cases, and the plate was removed in both cases. Revision surgeries of the internal fixation devices were performed, of which one case underwent autologous bone grafting at the osteotomy site.

### Radiographic evaluation

The preoperative WBLR (%) was 23.0 ± 15.72, which was significantly lower in the L Group (15.9 ± 13.9) than in the H Group (31.0 ± 18.6) (*p* < 0.028). The preoperative mMPTA, mLDFA, and KJLO were 83.8 ± 1.74, 87.7 ± 1.4 and 1.4 ± 1.7, respectively. The postoperative mMPTA and KJLO were 91.9 ± 1.95 and 1.9 ± 1.4, respectively. There were no significant differences between the two groups (Tables 2 and 3).

**Table 3:** Surgery‐related characteristics and clinical results.

Variables	Total
Preoperative KOOS(Sports/Rec)	**31.6** ± **17.8**
Preoperative Tegner activity score	3.9 ± 1.46
Postoperative KOOS(Sports/Rec)	47.2 ± 16.2
Postoperative Tegner activity score	4.2 ± 1.32
Flextion contracture (+) (knees)	14
Unstable hinge fracture (+) (knees)	2
Severe medial cartilage damage (+) (knees)^a^	29
Postoperative progression of OA changes in PF joint (knees)^b^	37

Abbreviations: ICRS, International Cartilage Repair Society; KOOS, Knee Injury and Osteoarthritis Outcome Score; MFC, medial femoral condyle; MTP, medial tibial plateau; OA, osteoarthritis; PF, patellofemoral.

^a^ Severe medial cartilage damage is defined as cartilage damage of ICRS classification 4 in either the MFC or MTP.

^b^ Postoperative progression of OA changes in PF joints is defined as arthroscopic findings demonstrating a deterioration in either the patella or trochlea from before to after surgery according to the ICRS classification.

### Clinical evaluation

The pre‐ and postoperative KOOS (Sports/Rec) were 31.6 ± 17.8 and 47.2 ± 16.2, respectively. The pre‐ and postoperative Tegner activity scores were 3.9 ± 1.46 and 4.2 ± 1.32, respectively. Both scores were significantly higher in the H Group (preoperative, 49.0 ± 6.8 and 5.4 ± 0.6, respectively; postoperative, 73.9 ± 7.5 and 5.8 ± 0.8, respectively) than in the L Group (preoperative, 17.0 ± 8.0 and 3.9 ± 1.5, respectively; postoperative, 21.4 ± 7.9 and 3.1 ± 0.5, respectively), while the number of cases with postoperative knee joint contracture was significantly lower in the H group (0/27) than in the L group (12/33). Unstable hinge fractures were observed in two cases in the L group, but not in the H group (Tables 2 and 3).

### Arthroscopic findings

**Figure 2:**
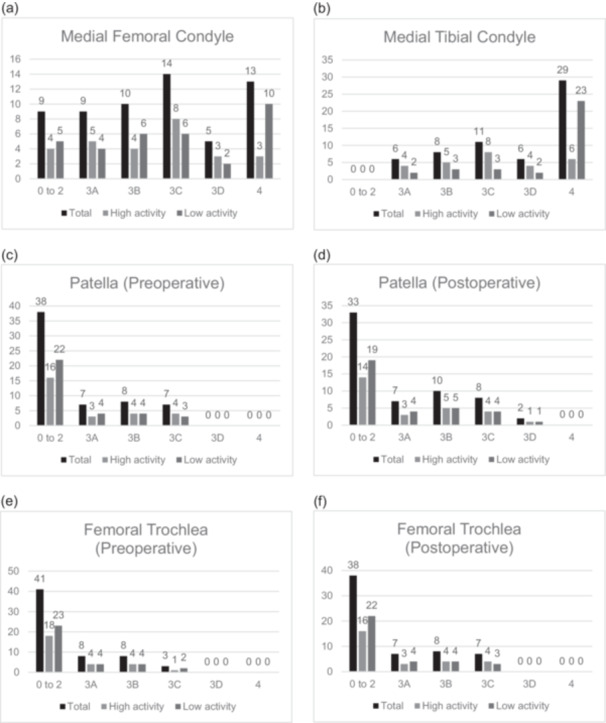
Cartilage damage in the medial compartment according to the ICRS classification. *Postoperative deterioration cases: total, 7; high activity, 3; low activity, 4. *postoperative deterioration cases: total, 35; high activity, 16; low activity, 19. ICRS, International Cartilage Repair Society.

Based on the ICRS classification, intraoperative arthroscopic findings of cartilage damage in the medial compartment revealed 47 cases with a medial femoral condyle (MFC) score of 3 or less (24 cases in the H Group and 23 cases in the L Group) and 13 with a total score of 4 (3 in the H Group and 10 in the L Group). In terms of the medial tibial condyle (MTP), there were 31 cases with a score of 3 or less (21 in the H Group, 10 in the L Group) and 29 with a total score of 4 (6 in the H Group and 23 in the L Group). The number of severe cartilage damages in the medial compartment before surgery (ICRS classification 4 in either MFC or MTP) was significantly lower in the H Group (6/27) than in the L Group (23/33) (Figure 2).

The number of OA changes in the PF joint at the time of implant removal (cases in which either the patella or trochlea worsened before or after arthroscopic examination in the ICRS classification) was 37/60 overall, 17/27 in the H Group, and 20/33 in the L Group, respectively, with no significant differences between the two groups (Table 4).

**Table 4:** Comparison of each factor between two groups.

Characteristics and variables	High activity return group (*n*＝27)	Low activityreturn group (*n*＝33)	*p*‐value
Age	51.9 ± 10.7	60.0 ± 7.6	**0.006** ^*^
Correction angle	8.9 ± 2.0	10.9 ± 1.7	**0.001** ^*^
Preoperative WBLR	31.0 ± 18.6	15.9 ± 13.9	**0.028** ^*^
Preoperative mMPTA	83.9 ± 2.5	83.5 ± 1.5	0.574
Preoperative mLDFA	87.7 ± 1.4	88.8 ± 3.0	0.252
Preoperative KJLO	1.1 ± 1.2	1.6 ± 2.1	0.387
Postoperative WBLR (%)	60.4 ± 5.4	57.6 ± 6.5	0.120
Postoperative mMPTA	91.4 ± 1.9	92.2 ± 1.9	0.163
Postoperative KJLO	1.6 ± 1.7	2.6 ± 2.3	0.387
Preoperative KOOS (Sports/Rec)	49.0 ± 6.8	17.0 ± 8.0	**0.00** ^*^
Preoperative Tegner activity score	5.4 ± 0.6	3.9 ± 1.5	**0.00** ^*^
Flexion contractures (+)	2/27 cases	12/33 cases	**0.00** ^*^
Unstable hinge fractures (+)	0/27 cases	2/33 cases	**0.146**
Severe cartilage damage (+)	6/27 cases	23/33 cases	**0.00** ^*^
Postoperative OA progression in the PF joint	17/27 cases	20/33 cases	0.99
Postoperative KOOS (Sports/Rec)	73.9 ± 7.5	21.4 ± 7.9	**0.00** ^*^
Postoperative Tegner activity score	5.8 ± 0.8	3.1 ± 0.5	**0.00** ^*^

Abbreviations: KJLO, knee joint line orientation; KOOS, Knee Injury and Osteoarthritis Outcome Score; mLDFA, mechanical lateral distal femoral angle; mMPTA, mechanical medial proximal tibial angle; OA, osteoarthritis; WBLR, weight‐bearing line ratio.

### Statistical analysis

Items that showed significant differences between the H Group and L Group were age, correction angle, preoperative WBLR, KOOS (Sports/Rec), preoperative Tegner activity score, presence of preoperative flexion contracture, presence of unstable hinge fracture, presence of severe cartilage damage, postoperative KOOS (Sports/Rec), and postoperative Tegner activity score. Factors influencing whether high physical activity could be achieved by logistic analysis were age, small correction angle, low preoperative WBLR, absence of severe medial cartilage damage, postoperative flexion contracture, and unstable hinge fracture (Table 5). In addition, the cutoff values for the Youden Index method via ROC analysis were a preoperative Tegner activity score of 4.0 and a preoperative KOOS (Sports/Rec) of 35.0. In cases with severe cartilage damage and cases with non‐severe cartilage damage, the preoperative and postoperative WBLR values that affect high‐activity return were measured using ROC analysis; however, no significant differences were observed in either case, and it was not possible to calculate a cutoff value.

**Table 5:** Factors influencing whether high physical activity can be achieved by logistic analysis.

Characteristics and variables	OR (95% CI)	*p*‐values
Age	1.102 (1.025–1.186)	*p* = 0.009
Correction angle	1.867 (1.224–2.847)	*p* = 0.004
Low preoperative %MA	1.076 (1.001–1.156)	*p* = 0.046
Complication of severe medial cartilage damage	18.33 (3.956–84.958)	*p* = 0.000
Complications of postoperative flexion contracture	35.556 (4.068–310.800)	*p* = 0.001

Abbreviations: CI, confidence interval; OR, odds ratio.

## DISCUSSION

The most important findings of this study were the presence of flexion, contracture, and severe cartilage damage to the medial component, among other indicators of preoperative activity that are poor prognostic factors for affecting return to sport. In addition, the results of this study also suggested that under‐collection should be avoided in cases with severe cartilage damage. The most commonly reported factor that affects return to physical activities is patient background. High preoperative activity, such as the presence of sports activity within one year before surgery, activity based on the Tegner score [9, 12, 13], and high motivation and expectations for return to physical activities have been reported as important factors [2, 32]. In addition, low BMI and young age have been reported as favourable factors for return to physical activities [7, 11]. In this study, young age, preoperative Tegner activity score, and KOOS (Sports/Rec), which indicate preoperative activity, were significantly higher in the H Group.

In particular, ROC analysis showed that if the preoperative KOOS (Sports/Rec) score was less than 35, return to physical activities with a Tegner 5 or higher was significantly more difficult, and among these, all cases with a running or jumping score of 0 exhibited difficulty returning to sport, which could potentially be used as an indicator for return to physical activities.

Flexion contracture has been reported to cause pain and stiffness during walking and movement [3, 29] and can increase the load on the contralateral knee [21]. It has also been reported that flexion contracture of > 5° can have a negative effect on subsequent degenerative changes to the patellofemoral joint [27]. In this study, even in cases with postoperative flexion contracture, pain during weight bearing originating from the medial compartment disappeared; however, pain persisted in the anterior knee, popliteal fossa, and iliotibial band, which may be factors that affect return to physical activities.

Early bone union and early weight‐bearing walking due to stable internal fixation of the osteotomy site has been previously reported to affect return to physical activities [33]. Unstable hinge fractures carry the risk of delayed bone union and pseudoarthrosis [22], suggesting that they may delay the timing of weight bearing and affect postoperative rehabilitation. In this study, the number of unstable hinge fractures was small and did not present a statistically significant difference; however, all cases were in the Non‐Return Group, and the presence of the fracture was considered a factor that adversely affects return to physical activities.

Nakayama et al. [25] reported that KL4 and an opening gap > 10 mm were risk factors for return to physical activities. Similar results were obtained in this study, as severe cartilage damage in the medial compartment and low preoperative WBLR (severe varus alignment) requiring a large correction angle were significantly higher in the non‐return to physical activities group.

The goal to achieve postoperative lower limb alignment is controversial. Many studies have recommended approximately 55% in WBLR [39], such as Laprade et al [17] at 54%–56%, De Neve et al. [4] at 53.4%, Maeda et al. [18] at 57.5%–62.5%, and Kanto et al. [12] at 51.6%. Overcorrection has also been reported to increase the load on the lateral compartment, increase the shear force on the cartilage due to unphysiological tibial plateau inclination [24, 38]. However, some studies have reported that insufficient correction leads to poor outcomes [11, 19, 20]. Feucht et al. [6] reported that it is desirable to perform an appropriate case according to the KL classification and stated that the goal is achieve a WBLR of 50–55 for KL Grade 0, 55–60 for Grades 1–2, and 60–65 for KL 3–4. In this study, the number of cases was small, and no statistically significant differences were observed; therefore, it was not possible to calculate a cutoff value for lower limb alignment. However, as a reference value, the cutoff value in cases with severe cartilage damage of ICRS 4 or higher in the medial compartment was a postoperative WBLR of 63.5%. Based on this, the under‐collection of cases with severe cartilage damage should be avoided.

The limitations of this study include the study sample that comprised of a single ethnic group, small number of cases, differences in treatment methods for arthroscopic meniscal tears that could influence outcomes, and short follow‐up period.

## CONCLUSION

The preoperative KOOS [Sports/Rec] subscore was a useful predictor of cases able to return to physical activities with a Tegner 5 or higher after OWHTO. Flexion contractures and unstable hinge fractures may be risk factors for achieving high physical activity after surgery; therefore, it is important to conduct range of motion exercises of the knee before and after surgery. In addition, precise surgical techniques are essential to prevent unstable hinge fractures. In cases with significant cartilage damage, care should be taken to avoid under‐collection.

## AUTHOR CONTRIBUTIONS

Fumiyoshi Kawashima wrote the initial draft of the manuscript. Jun Oike and Kazuyuki Segami contributed to the data collection and analysis. Koji Kanzaki supervised the work. All authors reviewed the final draft of the manuscript.

## CONFLICT OF INTEREST STATEMENT

The authors declare no conflicts of interest.

## ETHICS STATEMENT

This study was approved by the Ethics Committee of Showa University Fujigaoka Hospital (approval number: 2024‐248‐A). Patients signed informed consent regarding publishing their data and photographs.

## Data Availability

The authors agree to make data and materials supporting the results or analyses presented in their paper available upon reasonable request.
